# miRNAs Involvement in Modulating Signalling Pathways Involved in Ros-Mediated Oxidative Stress in Melanoma

**DOI:** 10.3390/antiox13111326

**Published:** 2024-10-30

**Authors:** José Daniel Escobar Moreno, José Luis Fajardo Castiblanco, Laura Camila Riaño Rodriguez, Paula Marcela Barrios Ospina, Carlos Andrés Zabala Bello, Esther Natalia Muñoz Roa, Hernán Mauricio Rivera Escobar

**Affiliations:** 1 Semillero de Investigación de Medicina (SIMED), Basic and Translational Research Group (GIBAT), Faculty of Medicine, Universidad El Bosque, Bogotá 110121, Colombia; jdescobarm@unbosque.edu.co (J.D.E.M.); jfajardoc@unbosque.edu.co (J.L.F.C.); lcriano@unbosque.edu.co (L.C.R.R.); pbarrios@unbosque.edu.co (P.M.B.O.); 2 Laboratory of Animal Cytogenetics, Faculty of Veterinary Medicine and Animal Science, Universidad Nacional de Colombia, Bogotá 111321, Colombia; caazabalabe@unal.edu.co; 3 PhD Program in Biological Sciences, Faculty of Science, Pontificia Universidad Javeriana, Bogotá 110231, Colombia; en.munozr@javeriana.edu.co; 4 Department of Interdisciplinary Studies—DEI, Instituto de Educación a Distancia—IDEAD, BIOPESA Research Group, University of Tolima, Ibagué 730006, Colombia

**Keywords:** melanoma, ROS, miRNAs, redox homeostasis, oxidative stress

## Abstract

Reactive oxygen species (ROS) are intermediates in oxidation–reduction reactions with the capacity to modify biomolecules and temporarily or permanently alter cell behaviour through signalling pathways under physiological and pathophysiological conditions where there is an imbalance between oxidative factors and the antioxidant response of the organism, a phenomenon known as oxidative stress. Evidence suggests that the differential modulation of ROS-mediated oxidative stress occurs in the pathogenesis and progression of melanoma, and that this imbalance in redox homeostasis appears to be functionally linked to microRNA (miRNA o miRs)-mediated non-mutational epigenetic reprogramming involving genes and transcription factors. The relationship between ROS-mediated stress control, tumour microenvironment, and miRNA expression in melanoma is not fully understood. The aim of this review is to analyse the involvement of miRNAs in the modulation of the signalling pathways involved in ROS-mediated oxidative stress in melanoma. It is hoped that these considerations will contribute to the understanding of the mechanisms associated with a potential epigenetic network regulation, where the modulation of oxidative stress is consolidated as a common factor in melanoma, and therefore, a potential footprint poorly documented.

## 1. Introduction

The term ROS refers to chemical structures such as superoxide anions, peroxides, and hydroxyl radicals which contain one or more unpaired electrons; they are intermediates in oxidation–reduction reactions with the capacity to modify biomolecules and temporarily or permanently alter cell behaviour through various signalling pathways in physiological and pathophysiological conditions where there is an imbalance between oxidative factors and the antioxidant response of the organism, a phenomenon known as oxidative stress [1]. While in a healthy cell, stress potentiates mechanisms that generally lead to cell death, in cancer, a strong survival capacity of tumour cells is observed at levels of oxidative stress increased by ROS, a condition that involves, among other things, genetic and epigenetic changes that affect the biochemistry and modulation of mitochondrial function [2,3].

Melanoma is a type of skin cancer that originates from melanocytes, cells specialised in melanin synthesis, and is characterised by increased cell proliferation, resistance to chemotherapeutic agents, and metastasis. According to the World Health Organization (WHO), melanoma is one of the deadliest cancers in the world, with a survival rate of more than 80% of the reported cases in the early stages of diagnosis, but falling to 5% in the metastatic stages [4]. The phenotypes associated with the pathogenesis and progression of this cancer include the modulation of oxidative stress, which coincides with changes in gene expression and metabolic reprogramming involving miRNAs; small non-coding RNAs involved in post-transcriptional epigenetic regulation, which regulate the cellular response to changes in the microenvironment by acting as switches in pathway activation; and gene repression in virtually all cancer pathways involved in adaptation and survival [5]. It is possible that this modulation of oxidative stress may emerge as a new hallmark underlying cancer.

The relationship between the control of ROS-mediated stress, the tumour microenvironment, and the expression of miRNAs in melanoma is not fully understood due to the use of linear regulatory models that limit the integration of dynamic molecular architecture and configuration associated with the modulation of oxidative stress as a survival strategy. Therefore, new approaches are required, possibly from network biology that integrates signalling pathways, transcription factors, miRNAs, and genes, which together will guide the development of new therapeutic strategies for early diagnosis and potentially effective treatments for more advanced stages of this cancer [6,7]. The aim of this review is to analyse the involvement of miRNAs in the modulation of signalling pathways in ROS-mediated oxidative stress environments in melanoma. The paper is divided into three sections: the first section deals with the described relationship between miRNAs and ROS in melanoma; the second section reviews the involvement of different signalling pathways modulated by ROS and their relationship with changes in miRNA expression; and the third section presents a bioinformatic and statistical functional enrichment approach for the construction of potential network models mentioned above based on a dataset reported in the Gene Expression Omnibus (GEO).

## 2. miRNAs, ROS and Melanoma

Molecular mechanisms associated with melanoma tumour physiology in processes such as proliferation, growth, energy metabolism, migration, differentiation, and cell death involve oxidative stress and thus the genetic and epigenetic alterations of oncogenes and tumour suppressor genes [6,8,9]. Non-mutational epigenetic reprogramming is exerted by miRNAs, small non-coding RNAs of 18–22 nt, which play a role in the post-transcriptional regulation of up to 60% of mammalian protein-coding messenger RNA (mRNAs) [6,10]. Most miRNAs are derived from long intramolecular double-stranded RNAs; these RNAs are sequentially cleaved by type III RNases, first in the nucleus and then in the cytoplasm, to generate a miRNA duplex. In the subsequent steps, one of the strands of the duplex associates with an RNA-induced silencing complex (RISC), which triggers a decrease in a specific mRNA by degrading the transcript or repressing mRNA-to-protein translation [11]. A single miRNA can target hundreds of mRNAs, and a single mRNA can target multiple miRNAs, so variations in the expression of thousands of mRNAs could be explained by the coordinated network expression patterns of a few miRNAs [12]. There are miRBase records of 2600 corresponding sequences for mature miRNAs in humans [13,14,15,16].

Potential relationships between ROS and miRNAs in cancer have been reported; e.g., miRNA-21 can inhibit the antioxidant pathway of superoxide dismutase (SOD), facilitating oxidative damage by superoxide anion (O^2−^) and other ROS, and enhance oncogenic and fibrotic processes associated with carcinoma [16,17]. Alterations in miRNA-5096 increase hydroxyl radical (OH^−^) levels and suppress non-apoptotic cell proliferation in breast cancer cells; the mechanism of cell damage of this miRNA is mediated by ROS and is reversible with the use of antioxidants such as N-acetyl cysteine (NAC) [18]. Meanwhile, it has been shown that the increase in miRNA-34 in human glioma cells, together with the suppression of nitrogen dioxide radicals, supports the increase in apoptosis rate and the decrease in cell viability in a glioma model [19].

In melanoma, miRNAs are also differentially expressed and their alteration may be related to changes in the oxidative environment and the activation of cancer imprints. For example, the enrichment of extracellular vesicles with miRNA-214 secreted by melanoma cells stimulates the over-activation of macrophages, which release nitric oxide (NO), a process that facilitates endothelial permeability and favours metastasis [11]. NO alone is not capable of causing DNA damage, but it can inhibit enzymes such as DNA ligase and thus indirectly cause double helix breaks [20]. It has also been suggested that silencing miRNA-517a in melanoma patient samples results in the overexpression of CDKN1C (cyclin-dependent kinase inhibitor 1C) and suppression of the c-Jun N-terminal kinase (JNK)-mediated survival and proliferation pathway, leading to increased ROS [7]. Furthermore, excessive levels of ROS, such as those caused by the administration of chemotherapeutic agents, induce the development of resistance mechanisms to the oxidative environment; such resistance has been studied in the human melanoma cell line A375, in which the activation of the mitogen-activated protein kinase (MAPK) pathway (RAS/MEK/ERK) and the transcription factors hypoxia-inducible factor 1 (HIF-1α) and the master regulator of melanocyte differentiation, microphthalmia-associated transcription factor (MITF), were observed.

Indeed, other miRNAs with different molecular targets have been linked to the HIF-1α and MITF pathways. The miR-33a and miR-138 are thought to directly regulate HIF-1α in the transition from melanocyte to metastatic melanoma and breast cancer [21,22]. miR-182 induces increased invasion and metastasis by binding to the tumour suppressor F-box/WD repeat-containing protein (FBXW), and its binding to FOXO3 and MITF appears to be associated with melanoma progression, and miR-182 is a target of epigenetic modulation with hypermethylated CpG islands in melanoma cells [14]. On the other hand, miR-211 modulates the expression of genes involved in cell cycle regulation and tumour suppression, such as p16INK4A, BRN2, and MITF [23,24]. Meanwhile, miR-203 has been suggested to act as a tumour suppressor by regulating melanosome transport and tyrosinase enzyme (TYR) expression through the kinesin 5 (kif5b) superfamily of proteins and appears to negatively modulate one of the major signalling pathways active in melanoma cells, the CREB1/MITF/Rab27a pathway [25].

In addition to promoting a tumorigenic environment, miRNAs can also act as regulators of p53-associated tumour suppressor conditions and molecular machinery [26,27,28,29]. The activation of p53, stimulated by cellular stresses such as ionising radiation, hypoxia, carcinogens, or oxidative stress, leads to cell cycle arrest and promotes DNA repair or induces apoptosis through various pathways [30]. The expression of miR-18b is significantly reduced in patient-derived melanomas and cell lines due to the hypermethylation of the p53 pathway, while its stable overexpression results in potent tumour suppressor activity as measured by cell viability, the induction of apoptosis, and reduction in tumour growth in xenograft assays [31].

It is possible that oxidative stress in melanoma involves non-mutational epigenetic reprogramming in which networked sets of miRNAs, together with transcription factors such as MITF and HIF-1α, exert control over specific mRNAs whose protein products modulate the activity of various signalling pathways that ensure survival under these stress conditions, an aspect that will be discussed in the next section.

## 3. Oxidative Stress Signalling Pathways and miRNA Expression in Melanoma

Growth suppressor evasion, resistance to cell death, replicative immortality, angiogenesis, invasiveness, metastasis, and sustained proliferative signalling are hallmarks of cancer, coupled with energy dysregulation, genomic instability, the release of phenotypic plasticity, non-mutational epigenetic reprogramming, and sustained inflammation, with a characteristic stress-mediated environment [32]. These hallmarks coincide with the modulation of several melanoma-associated signalling pathways, such as the MAPK pathway and MITF, which stimulate growth. There is also the activation of the phosphatidylinositol 3-kinase (PI3K/AKT) pathway, which is responsible for altering extracellular functions as a metabolic regulator of survival and a generator of cell growth signals [33,34].

HIF-1α also acts as a transcriptional mediator of tissue hypoxia; HIF-1α is hyperactivated in conditions of prolonged anoxia as a consequence of possible adaptation to tumour microenvironment factors, the reduction in mitochondrial respiration in low-oxygen environments, and the subsequent Warburg effect, conditions that alternate with the control of ROS and therefore the modulation of oxidative stress. Specifically, HIF-1α inactivates the enzyme pyruvate kinase, thereby redirecting cellular metabolism towards glycolysis [35]. Mutations in signalling pathways such as PI3K/AKT can stabilise HIF-1α even under normoxic conditions [35,36]. Other signalling pathways, such as NRF2/Keap1, regulate the antioxidant response in melanoma and have an ambivalent function, protecting the healthy melanocyte from stress but also promoting tumour cell survival against ROS-induced damage [37]. Other ROS-enriched environments may promote pathways such as the Wnt pathway, which is involved in melanogenesis through MITF [38]. Signalling interactions are not necessarily linear, but involve a complex multi-scale regulatory network that can be reprogrammed by miRNAs even in the presence of genetic changes [39].

### 3.1. MITF Signalling Pathway

Studies have linked MITF function to cellular plasticity in melanoma, and several changes in its expression and activity as a suppressor and mediator of tumour progression have been documented [40]. These regulatory mechanisms include cellular extrinsic signals in combination with intrinsic post-transcriptional epigenetic changes that determine the status of tumour cells [41]. The microenvironmental signals generated drive a reversible phenotypic switch between a proliferative and a highly invasive phenotype.

Melanocytes are melanin-producing cells whose differentiation, proliferation, and survival are highly dependent on MITF [42]. In normal cells, MIFT is critical for melanocyte survival through the increased expression of B-cell lymphoma 2 (BCL2) and BcL xL (anti-apoptotic factors) and DICER, a factor that has been shown to be critical for melanocyte survival through its role in processing miRNAs [43]. In melanoma cells, the depletion of these anti-apoptotic proteins, particularly Bcl-2, leads to cell death [42].

The expression of MITF is regulated by melanocyte-stimulating hormone (α-MSH) and its action on the MC1R receptor; this interaction increases the transcription of genes related to pigmentation in terms of melanin synthesis. The physiological response requires exposure to ultraviolet radiation (UVR), which increases the likelihood of DNA damage and activation of p53, leading to the transcription of pro-opiomelanocortin (POMC) in keratinocytes. POMC is enzymatically cleaved to produce Alpha-melanocyte-stimulating hormone (αMSH) which binds to MC1R in melanocytes. This triggers the rest of the pigment response, resulting in the transcription of MITF and its targets TYR, dopachrome tautomerase (DCT), and tyrosinase-related enzyme 1 (TYRP1). Melanin is produced in melanosomes and transported to keratinocytes, where they form protective layers over their nuclei [44].

The transcriptional control of MITF is provided by a number of transcription factors and regulators associated with signalling pathways such as LEF1 (lymphoid enhancer-binding factor 1) and β-catenin, both effectors of the Wnt pathway, discussed below [41]. It has also been shown that this αMSH/MC1R/cAMP/PKA signalling cascade can direct β-catenin to specific cAMP response element-binding protein (CREB) promoters to activate the transcription of target genes, including MITF [45]. Zinc finger E-box binding homeobox 1 and 2 (ZEB1–ZEB2) are genes that determine cellular state by modulating MITF expression. ZEB2 promotes MITF expression and is associated with a differentiated and proliferative cell state. In addition, ZEB1 is associated with low MITF expression and a more invasive cell state [46].

In addition to the transcription factors and post-translational regulators described above, MITF regulation also involves the regulation of several miRNAs. In fact, using TargetScan 7.2, more than 30 potential sites in the 3′UTR region of the MITF gene (ENST00000328528.6) were found to be regulated by miRNAs [47]. The miRNA-182 promotes cell migration and survival in melanoma through the negative regulation of MITF [48]. It has also been reported that miRNA-137 harbours a melanoma susceptibility allele and is a down-regulator of MITF expression, apparently associated with cell cycle arrest in G1 [49]. The down-regulation of Bcl-2 expression by small interfering RNA has been reported to modulate miRNA-211 expression [50,51]. In addition, immunofluorescence assays revealed a reduction in MITF following Bcl-2 overexpression, suggesting that Bcl-2 may be a regulator of MITF in the context of melanoma [52]. Indeed, miR-211, a melanocyte lineage-specific miRNA, localises to an intron of Transient receptor potential cation channel subfamily M member 1 (TRPM1), a target gene of MITF, which would explain the above findings [51].

### 3.2. HIF-1α Signalling Pathway

Under normoxic physiological conditions, HIF-1α is regulated by its hydroxylation at proline residues for the subsequent binding to the von Hippel–Lindau tumour suppressor protein (pVHL), which mediates its degradation by the ubiquitin–proteosome complex [53]. A fundamental premise of the hyperactivation of the HIF-1α pathway under normoxic conditions is the sustained stimulation of growth signalling pathways such as PI3K/AKT, mTOR, Wnt/β-catenin, and NOTCH. Meanwhile, the HIF-1α pathway, which is activated under anoxic conditions and is sensitive to ROS, regulates the expression of miRNA-210, miRNA-421, miRNA-382, and miRNA-687, miRNAs with abnormal expression in cancer [54,55,56]. This suggests a link between the ROS activation of HIF-1α and abnormal expression of miRNAs associated with tumour progression. This phenotype may also occur in melanoma, where miRNA-mediated changes in HIF-1α and MITF expression have been reported [57,58].

αMSH regulates HIF-1α expression through cAMP. Given this specificity, it is likely that there are specific molecular mechanisms responsible for HIF-1α regulation in melanoma cells involving the previously described MITF signalling cascade. Using chromatin immunoprecipitation assays, it was concluded that MITF is a transcriptional target of HIF-1α through stimulation by cAMP [58]. Furthermore, in vivo and in vitro hypoxia signalling was identified as a negative regulator of MITF expression by finding that HIF-1α and MITF signalling were mostly closely correlated [57].

Several studies establish a clear relationship between some miRNAs and the HIF-1α signalling pathway in melanoma [59]. In 2015, Zhou et al. overexpressed miRNA-33a in the A375 cell line (amelanotic and metastatic) and in parallel inhibited the expression of this miRNA in the non-metastatic WM35 and metastatic WM45 cell lines; in this study, the authors demonstrated that miRNA-33a can inhibit the proliferation, invasion, and metastasis of human melanoma cells via the inhibition of HIF-1α. Similarly, in mouse experiments, miRNA-33a was shown to inhibit both the growth and metastasis of melanoma xenografts. These results provide a novel experimental basis for miRNA-33a to act as a tumour suppressor by the inhibition of HIF-1α in human melanoma cell lines [21]. In 2019, Qiu et al. performed a study in A375 and WM35 cell lines and found that miRNA-138 levels were negatively correlated with HIF-1α mRNA levels and that in cell tissues with advanced metastatic stages, there was a decrease in miRNA-138 along with a corresponding increased expression of HIF-1α activity with an apparent antagonistic function. Reducing HIF-1α activity by RNA interference inhibited E-cadherin expression, increased vimentin levels, and appeared to reverse the epithelial-to-mesenchymal transition process [60].

### 3.3. MAPK Signalling Pathway

One of the most important signalling pathways in melanoma is the MAPK pathway, a pathway that centralises aerobic cellular metabolism and affects mitochondrial metabolism where there is an accumulation of reducing species such as NADH; it is also an activator of ERK kinase and promotes the expression of MITF via MEK [61]. Different activating mutations have been identified in the melanoma subtypes of molecules that are part of this pathway, such as N-RAS, BRAF, MEK, and ERK; in fact, more than half of the reported melanoma mutations correspond to BRAF V600E [62,63].

Under physiological conditions, once the GDP-GTP exchange with the RAS protein occurs, its conversion to its active form is promoted, the PI3K/AKT pathway is potentiated, and the activity of the serine/threonine kinase RAF is stimulated, which phosphorylates and activates the tyrosine/threonine kinases MEK1 and MEK2 [64]. Next, MEK activates ERK1 and ERK2, then ERK translocates to the nucleus and activates cyclin CCND1, which forms the CCND1-CDK4/6 complex responsible for phosphorylating the RB1 protein, which in turn inactivates E2F, a growth factor in cell proliferation and metabolism [65,66].

As mentioned above, mutations in the BRAF gene occur in 50–60% of all melanomas, of which approximately 90% are V600E [67]; in this mutation, the amino acid glutamic acid (E) is substituted by valine (V) at position 600 of the protein [68]. In BRAF V600E mutations, there is increased kinase activity due to the lack of physiological negative feedback, leading to the permanent activation of the MAPK pathway. The MAPK signalling cascade can also be hyperactivated by defects in the p16 tumour suppressor, which is responsible for the negative feedback of CCND1, leading to the loss of negative regulation of retinoblastoma protein (RB1) phosphorylation and hence the release of E2F mentioned above [66]. The characteristics of the MAPK pathway also include the down-regulation of MITF activity through the ubiquitin–proteasome system. However, due to the subsequent phosphorylation of p38, the MEK cascade can promote MITF expression leading to melanocyte differentiation and thus pathological melanogenesis.

The MAPK pathway has been described to be affected not only by ligand–receptor interactions but also by various stressors in the cell. Oxidative stress caused by ROS can induce the activation of this pathway by ERK, JNK, or p38, but the mechanisms by which ROS can activate these kinases are not well defined [69]. Evidence in cancer suggests that oxidative stress induces the activation of an epidermal growth factor (EGF) receptor via the phosphorylation of receptor tyrosine kinases (RTKs), of which hydrogen peroxide is a mediator for independent phosphorylation. Some approaches focus on the molecular mechanisms underlying JNK and p38 activation by redox-sensitive proteins such as thioredoxin and glutaredoxin, following the link between the ability of ROS to oxidise thioredoxin to dissociate from ASK-1, leading to the subsequent activation of the JNK and p38 pathways [70].

The involvement of the MAPK pathway in processes such as proliferation, invasion, and migration in melanoma cells in parallel with the expression of specific miRNAs has been reported in several studies, and it has been suggested that miRNA-340 is able to regulate MAPK signalling by reducing the expression of phosphorylated Erk1/2. This suggests that miRNA-340 appears to be a modulator of this pathway [71]. There is also evidence that oncogenic MAPK signalling stimulates the miRNA-29 family, specifically p53-dependent miRNA-29b2/c transcription; however, it has also been proposed that levels of this miRNA decrease during melanoma progression. miRNA-29 and MAPK could act as tumour suppressors by targeting MAFG and MYBL2 [72]. At present, the role of miRNA-29 is not clear, although it could act as a tumour suppressor as its expression decreases in parallel with tumour progression, but it could also be a possible oncogene, being stimulated by MAPK and playing a role in melanogenesis [72].

### 3.4. PI3K-AKT Signalling Pathway

The PI3K/AKT signalling pathway is involved in cell survival and metabolic regulation. The hyperactivation of the pathway is present in more than 50% of melanomas as a result of AKT3 amplification and the subsequent loss of the tumour suppressor PTEN through epigenetic silencing or deletion [73]. AKT signalling is initiated by PI3K upon stimulation by exogenous growth factors, together with PIP expression, which promotes the translocation of AKT to the plasma membrane for activation by phosphorylation. The intracellular level of PIP3 is negatively regulated by the PTEN phosphatase, and the deficiency of this tumour suppressor induces the positive regulation of PIP3 and promotes AKT activation. Studies using RNA interference targeting AKT3 or PTEN mutations reduced the tumorigenic potential of melanoma cells [33]. It is possible that increased cellular metabolism may affect the production of free radicals, which cells then use as signalling molecules to regulate the MAPK and PI3K-AKT pathways to adapt to increased ROS levels and the subsequent oxidative stress.

Tumour cells can positively regulate the KEAP1/NRF2 pathway, a key pathway for sensing and responding to oxidative stress. Thus, high levels of ROS and antioxidant defence pathways are present in the cancer cell to ensure its survival and metabolic needs [34]. Evidence in cancer suggests that hydrogen peroxide-induced oxidative stress activates a PIP3-dependent signalling mechanism through the inactivation of the previously described tumour suppressor PTEN [74].

Previous studies have shown that miRNA-425 is able to inhibit cell proliferation by inducing apoptosis in A375 and SK-MEL-28 cells. Subsequent luciferase assays together with bioinformatics analysis have identified IGF-1 as a target gene of miRNA-425, which in turn inhibits the IGF-1-activated PI3K-AKT pathway. This suggests that miRNA-425 is able to inhibit melanoma progression through IGF-1 [75]. PCB2 is an oncogene that promotes tumorigenesis and metastasis; experimental evidence shows that in melanoma there is a negative regulation of miRNA-5195-3p and a positive regulation of PCBP2. It has been shown that miRNA-5195-3p inhibits PI3K/AKT activation in melanoma by inhibiting this gene [76].

### 3.5. Wnt Signalling Pathway

The Wnt signalling pathway plays a fundamental role in the differentiation and proliferation of melanocytes in the embryonic stage and in colon, breast, and prostate cancer [38]. In melanoma, its role is controversial because the levels of the pathway mediator, β-catenin, decrease as cell proliferation progresses in an inverse relationship [77]. This pathway is activated by a canonical axis linked to the growth and transformation of melanoma cells [48] through the specific binding of Wnt1a and 3a proteins to the FZD (Frizzled)/LRP (low-density lipoprotein receptor) receptor complex, which, after anchoring to the destruction complex formed by the Axin protein, Adenomatous polyposis coli (APC), glycogen synthase kinase-3 (GSK-3), and casein kinase-1 (CK1), allows β-catenin to be released into the nucleus, where it acts as a coactivator in the TCF (T-cell transcription factor/LEF (lymphocyte enhancer factor) complex, regulating the expression of the genes involved in cell proliferation [77]. It has even been shown that β-catenin itself is a regulator, as is the MAPK pathway acting on MITF, which is fundamental in controlling cell proliferation, survival, and differentiation in melanoma.

The non-canonical axis is associated with metastasis and acts independently of β-catenin. Wnt5a forms a receptor complex with FZD that activates JNK/PKC and calcium, which can transactivate Jun and nuclear factor of activated T-cells (NFAT) response genes involved in cell cycle regulation and contribute to survival. Similarly, the Wnt a5 ligand can down-regulate the lymphoid enhancer-binding factor LEF-1 by favouring cell invasion through the retention of β-catenin and promoting its degradation by ubiquitination through the up-regulation of Siah2 [78].

The association of this pathway with various miRNAs in processes such as cell proliferation, migration, and apoptosis in melanoma has been investigated. Inhibition of miR-10b reduced melanoma cell proliferation, migration, and invasion in vitro and tumor growth in vivo. ITCH (E3 Ubiquitin Protein Ligase) was identified as a direct target of miR-10b, influencing melanoma progression by regulating the Wnt/β-catenin pathway [79]. Oncomirs involved in the positive regulation of Wnt have also been reported, such as miRNA-25, which was overexpressed in the MV3 cell line and down-regulated the tumour suppressor and Wnt regulator DKK3 [80]. On the other hand, Wnt suppression affects tumorigenesis; Shi and his group proposed in 2019 that miRNA-22 targets FMNL2, a gene whose protein would be related to metastasis and tumour progression, and by inhibiting it suppresses melanoma development and down-regulates Wnt [81].

The positive regulation of Wnt is not only due to the expression of specific miRNAs, but also to the presence of an environment with increased oxidative stress from ROS. Wu and co-workers suggest that Wnt activation requires NOX1, a protein that promotes an oxidative stress environment and inhibits nucleoredoxin, a suppressor of the Wnt pathway [82], suggesting that the activation of the Wnt pathway is stimulated by ROS and by the expression of specific miRNAs that promote cell survival and proliferation in melanoma.

### 3.6. NRF2/KEAP1 Wnt Signalling Pathway

In melanoma development, proliferation, invasion, and survival, there is evidence for the increased expression of erythroid nuclear-related factor 2 (NRF2) [83], a fundamental player in the antioxidant response that ensures tumour survival to ROS damage [37]. Under physiological conditions, it is negatively regulated by Kelch-like ECH-associated protein 1 (KEAP) to maintain its basal level in the cytoplasm through degradation by the ubiquitin–proteosome system [83]. This regulatory protein has three characteristic domains in which IVR triggers the response after sensing redox reactions by oxidising, allowing NRF2 to dissociate from the regulatory complex and migrate to the nucleus where it dimerises with the small protein homologue of the aponeurotic muscle fibrosarcoma oncogene (sMaf). This heterodimer then binds to the antioxidant response element (ARE) within the DNA, triggering transcription at target genes and the recruitment of other transcriptional activators [83].

However, other mechanisms are known by which NRF2 contributes to melanogenesis, as it inhibits MITF, developing dedifferentiated and invasive melanoma [84], and exerts a function on the redox capacity of melanoma, being more expressed in advanced, metastatic, and drug-resistant melanoma by nuclear accumulation without affecting the Kelch-like ECH-associated protein 1 (KEAP1) levels in the cytoplasm, performing a positive regulation of NRF2 target genes [85]. A study in A375 cells and G361 cells showed that non-thermal plasma (NTP)-induced cell damage and caspase activity in G361 cells decreased in the presence of cytoglobin through the activation of the NRF2 pathway, suggesting that cytoglobin expression and the presence of melanin increased the resistance of melanotic melanoma cells to oxidative stress damage by the activation of antioxidant systems [85]. Nrf2/Keap1 upon exposure to ROS leads to the translocation of Nrf2 to the nucleus, resulting in an increase in antioxidant gene expression and thus a decrease in ROS levels [86,87].

The role of the NRF2/KEAP1 pathway in melanoma development is twofold: on the one hand, its potential antioxidant function reduces the likelihood of cancer; however, there is evidence that this pathway is also used as a mechanism of resistance to ROS damage in melanoma. The pathway is known to be associated with the expression of miRNAs identified as potential tumour suppressors such as miRNA-29, miRNA-181c, and miRNA-200c, but also with the expression of potential oncogenes such as miRNA-193b-365, miRNA-32, and miRNA-592 [88].

## 4. Expression Networks and Regulation under Oxidative Stress-Induced Melanoma

The study of signalling pathways provides insight into the molecular mechanisms involved in melanoma development and progression, including alterations in ROS-induced oxidative stress. However, more recent approaches integrate functional enrichment by downstream signalling pathways into the construction of gene regulatory networks between miRNAs, genes, and transcription factors, facilitating the abstraction of information in a visual format that is often intuitive and interpretable of non-linear regulation exerted by miRNAs [89,90]. As an example of this approach, regulatory and coexpression networks were constructed from the GSE109378 dataset reported in GEO [91], in which changes in gene expression were evaluated by next-generation sequencing (NGS) in the human melanoma cell line (SKMEL28), with the silencing of miR-211 (SK-P8-2) and xenografts in each line, and their potential association with changes in ROS levels, mitochondrial respiration, and cell growth and invasion processes [91].

**Figure 1:**
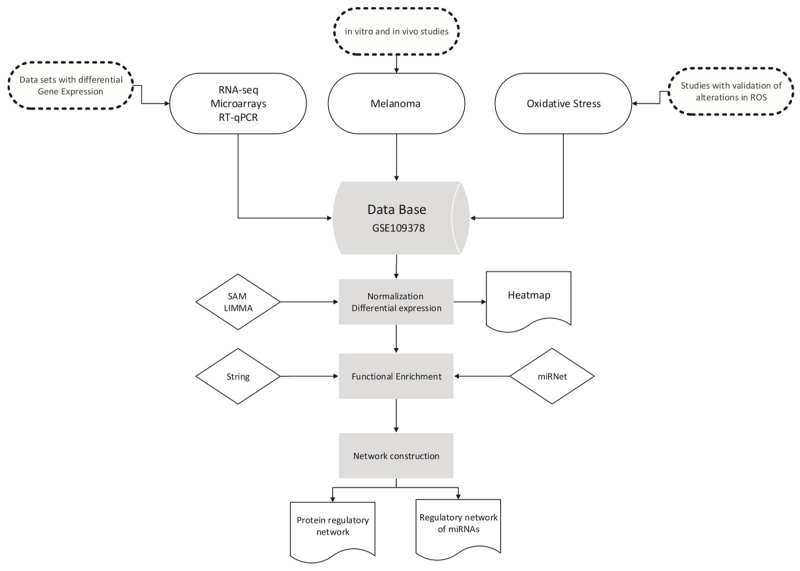
Workflow for database selection and graph construction.

From these data, differential expression analysis was performed using the SAM and LIMMA libraries (Bioconductor) in R code [92,93]. The data were normalised in the VSN library (Bioconductor) by stabilising the means and making the samples comparable using a Log2 transformation. For log-fold change estimation, the expression of those mRNAs with a *p*-value, corrected for multiple testing, of less than 5% was defined as differential [93]. The comparison was first performed between the SKMEL28 and SK-P8-2 lines and between the respective xenografts, and then the differentially expressed mRNAs were compared between the lines and xenografts (a total of six samples) (Figure 1). Initially, 1300 mRNAs with differential expression were obtained and, after manual curation, 30 of them were related to oxidative stress processes; finally, 6 were found to be differentially expressed. The intensity values of the mRNAs were visualised in heatmaps using the “pheatmap” function in the R code, taking the average value for each of the selected mRNAs in the cell line samples (cell line) and xenograft samples (Xeno). The colour and intensity of the squares were used to represent changes (absolute values) in expression [94].

**Figure 2:**
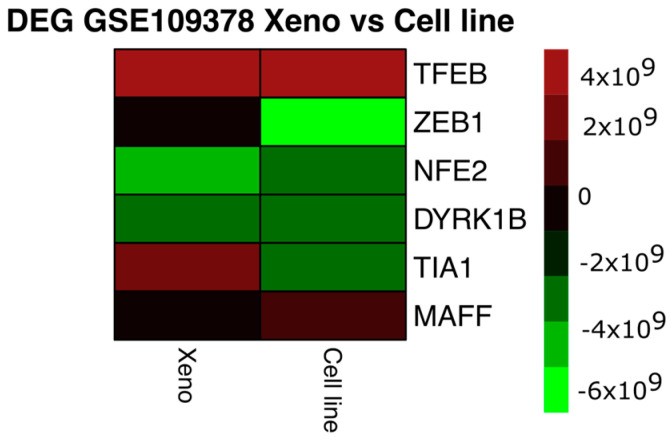
Heatmap of differentially expressed genes (DEGs) between the cell line depleted of miR-211 and the xenografts derived from these cell lines. The colour and intensity of the squares represent changes (absolute values) in expression. Red (over expression) and green (under expression).

As shown in Figure 2, MAFF and TFEB mRNAs are up-regulated in both the cell line and Xeno samples. MAFF mRNA has been implicated in melanogenesis by forming a heterodimer with NRF3 in response to stress [95], whereas the TFEB gene would cross-regulate with MITF in cellular clearance pathways [96,97] and induce melanoma growth by participating in metabolic regulation and ERK1/2 activation [98]. ZEB1, NFE2, and DYRK1B mRNAs were found to be decreased in both conditions. The transcription factor ZEB1 is a known inducer of mesenchymal–epithelial transition and invasiveness; in melanoma, elevated ZEB1 levels are associated with resistance to treatment with MAPK inhibitors [99], while the ZEB1/ZEB2 ratio would mediate phenotypic plasticity [46,99,100]. The transcription factor NFE2 is involved in megakaryocyte production [101]; although it has not been reported to be altered in melanoma, its regulation by Nrf2 would promote the accumulation of ROS in megakaryocytes [102].

Dual-specificity tyrosine phosphorylation-regulated kinase 1B-DYRK1B is activated by the RAF-MEK1/2-ERK1/2 signalling pathway in melanoma and would be related to the promotion of cell differentiation [103]. The only gene that showed differential expression in the conditions studied was the T-cell intracellular antigen 1 gene, which encodes the TIA1 protein, an RNA-binding protein associated with nucleolytic activity against cytotoxic lymphocyte target cells, which has been proposed to be an oncogene localised in the cytoplasm of oesophageal squamous cell carcinoma cells and to promote the expression of the progression-related genes SKP2 and CCNA2 [104]. It is possible that the increase in TIA1 in the xenografts derived from the miR-211 deletion lines compared to deletion-only cell lines reflects the influence of media-influenced tumour growth on the expression of tumour progression-related genes.

**Figure 3:**
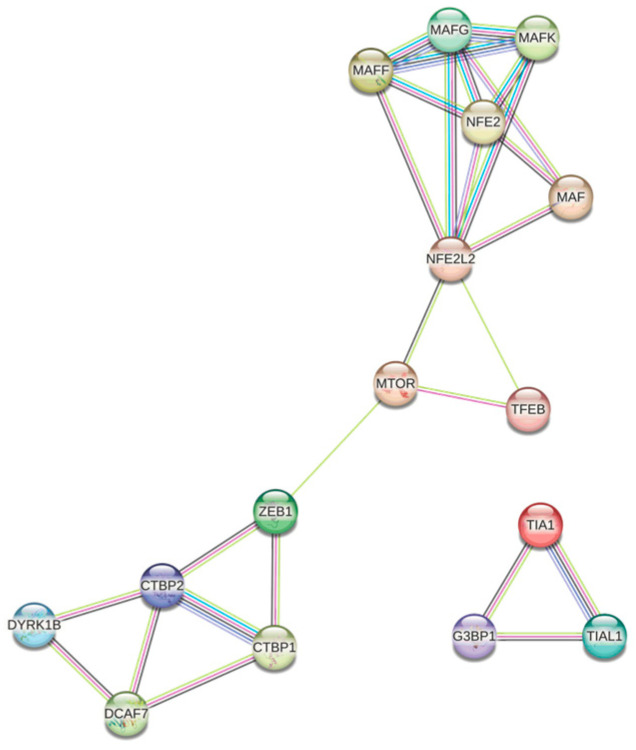
Protein regulatory network associated with oxidative stress in melanoma.

Using the String platform, the coexpression network was constructed (Figure 3), where the proteins encoded by the genes over- and underexpressed in both conditions are related to the signalling pathways associated with oxidative stress, response to ROS, response to cytoplasmic stress, and some of the pathways mentioned above, such as the PI3K-AKT-mTOR signalling pathway.

Figure 3 shows several distinct nodes. The node shown in blue/purple, formed by TIA1, G3BP1, and TIAL1, is associated with the formation of stress granules (SGs), cytoplasmic structures induced in response to environmental stress, mainly viral infection [105]; the formation of SGs appears to play an important role in the progression of several types of cancer by inhibiting apoptosis in response to stress [106,107]. Although in melanoma the presence of the retinoblastoma binding (RB) mRNA Rbfox2 in SGs was associated with progression and metastasis [108], the presence of TIA1, G3BP1, and TIAL1 in melanoma-derived SGs has not been reported, so new experimental approaches would be needed.

Another highly interconnected node is the one shown in white, formed by DYRK1B, DCAF7, and CTBP1/2. DCAF7 and DYRK1B form a complex associated with the regulation of processes such as cell proliferation, differentiation, and survival; it has been proposed that DCAF7 acts as an adaptor protein capable of mediating the binding and subsequent phosphorylation of E1A by DYRK1A/B, triggering a suppressive effect on proliferation through the negative regulation of the transcriptional co-repressor CTBP [109]. In addition, elevated levels of CTBP1 in melanoma have been associated with increased proliferation and DNA damage [110]; although by sequencing CTBP1 mRNA was not found to be differentially expressed in the two conditions evaluated, it is possible that by qPCR, differences related to the decrease in DYRK1B could be found (Figure 3).

The most highly connected node contains the proteins NFE2, MAF, and NFE2L2, which have been implicated in chaperone-mediated autophagy, a process of the selective degradation of cytosolic proteins in response to oxidative stress [111]. It has been reported that NRF2 has the function of regulating the antioxidant response, either by promoting tumour resistance to oxidative stress damage or by modulating carcinogenesis [97], while MAFF has been evaluated as a tumour suppressor and its interaction with NRF2 may suggest a protective antioxidant response of the healthy melanocyte.

Finally, proteins associated with PI3K-AKT-mTOR are shown in pink. In 2015, Hambright et al. demonstrated in different cell lines the key role of PI3K/AKT/mTOR signalling and the up-regulation of the antioxidant system in ensuring the survival of melanoma cells. Altering redox homeostasis by increasing oxidative stress in melanoma cells inhibited PI3K/AKT/mTOR signalling by disrupting mTORC1 formation, thereby reducing colony formation and cell proliferation [34]. However, the interaction between mTOR, ROS, and the antioxidant response is not fully understood, as these ROS may play a dual role by possessing both activating and inhibitory functions [112].

For the construction of regulatory network models, we used miRNet “https://www.mirnet.ca/ (accessed on 7 July 2024)”, a freely available network-based web tool that integrates several statistical tools, data mining, and visualisation systems for the integrated study of miRNA-molecular target interaction [94]. In addition to implementing a flexible interface for filtering, refining, and customising data during network construction, miRNet includes a network visualisation system with the possibility of functional enrichment analysis. The tool was fed with the IDs of the over- and underexpressed mRNAs from Figure 1 and for functional enrichment, the KEGG and GO pathways were used with the statistical analysis of a hypergeometric test.

**Figure 4:**
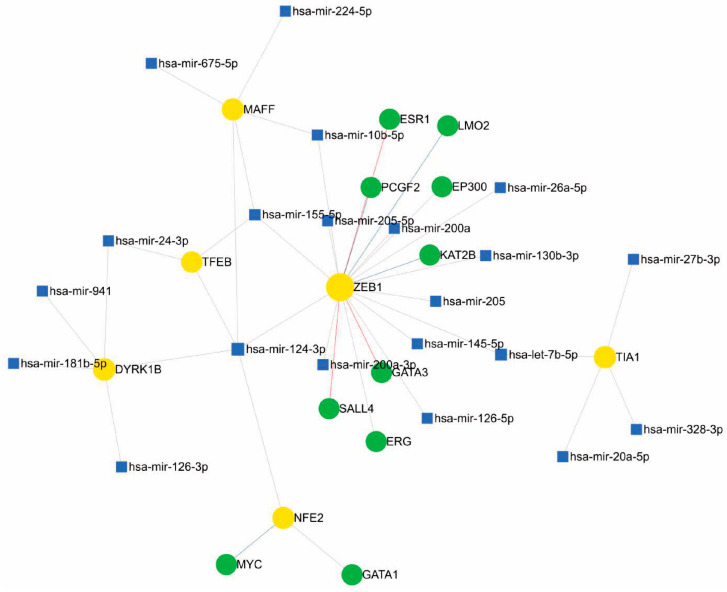
Regulatory network of miRNAs, genes, and transcription factors associated with oxidative stress in melanoma.

In Figure 4, the transcription factor ZEB1 (zinc-fingered E-box binding homeobox 1), best known for its involvement in mesenchymal–epithelial transition processes, cancer cell differentiation, progression, and metastasis [45,113] through the regulation of proteins such as SALL4 and GATA3 [114,115], which were also found to be related in the network, is the hub with the highest number of interactions. Its involvement in modulating the oxidative stress environment has been described in breast cancer models through the promotion of MCT4 and GPX4 [116,117]; in melanoma, it has been associated with increased resistance to MAPK inhibitors, promoting immune evasion [99,118]. It is possible that the highly defined DNA- and protein-binding domains explain the large number of interactions represented in the hub and the high modularity exerted. MAFF has been postulated as a possible tumour suppressor regulated by miRNA-224-5p in hepatocellular carcinoma [119].

Among the miRNAs that potentially modulate ZEB1 in the proposed network, we can highlight some that have been reported in the literature to act on other molecular targets in melanoma. The miR-126 inhibits invasion and migration in cervical cancer cells by binding to ZEB1 [104]; the miR-126-3p isoform was found to be associated with the transcription factor DYRK1B, and is involved in acquired resistance to dabrafenib in melanoma cells by regulating ADAM9 and VEGF-A [120]. The miR-200a-3p has been reported as a potential regulator of melanogenesis through direct binding to MITF [121]. miR-205-5p functions as a negative suppressor of the PI3K/AKT pathway in renal cancer [120]. Another node described is miR-224-5p and MAFF. This miRNA has been reported as an oncomiR and biomarker in several types of cancer [122,123]; additionally, miR-224-5p binding to MAFF occurs in hepatocellular carcinoma [119].

In the network, one of the miRNAs that interact with multiple targets is miR-24-3p, which has been associated with negative regulation in metastatic cancer; in B16F10 melanoma cells, ectopic expression could be generated in melanoma, which would generate suppression in cell migration. miR-24-3p generates the suppression of cancer cell growth, and the overexpression of this miR-24-3p could generate a decrease in cell viability, as well as the inhibition of cell migration and invasion. miR-24-3p could control cell proliferation by regulating hydrofolate reductase [124].

Meanwhile, exosomal miR-155-5p would be related to the pro-angiogenic onset of Caf (cancer-associated fibroblasts), as it could be delivered to fibroblasts and generate angiogenic factors such as vascular endothelial growth factor and fibroblast growth factor 2. The B16F10 cell line, which is a metastatic melanoma line, produces exosomal secretions with the ability to induce the reprogramming of fibroblasts into Caf and the expression of tumour angiogenesis markers. These exosomes suppress SOSC1 expression, leading to the activation of the JAK2/STAT3 pathway, which in turn regulates a pathogenic switch that increases vascular endothelial growth factor and fibroblast growth factor 2 expression in fibroblasts [125].

Likewise, TIA1 is modulated by miR-27b, among others, which is widely associated with melanoma development, as described in 2021 by Yi Tian and collaborators, where they determined expression levels in melanoma cells and normal tissues by immunoelectrotransfer, correlating a negative regulation with MYC, a gene related to the Wnt-β catenin pathway—a pathway of importance in melanogenesis described previously—as a target in this case [126]. Another modulator detected in the network is miR-20a-5p, also described in recent years as a tumour suppressor in different tissues, demonstrated in 2019 by Ahred and his team in B16 melanoma cells, where a suppression of the same was detected in comparison with non-malignant keratinocytes [126].

Although network approaches provide a deeper understanding of biological phenomena, it is essential to advance transcriptomic studies that enhance the available information, especially given the limited amount of data reported in the current databases. In recent years, sequencing technologies have advanced significantly from sequencing that enables single-cell transcriptomic profiling to spatial transcriptomics that provides information on the location of transcripts in histological sections. However, despite these advances, these technologies still have limitations in accurately quantifying miRNAs [127].

This review has highlighted the crucial role of reactive oxygen species and miRNAs in the modulation of oxidative stress and their relevance in the pathogenesis of melanoma. The redox imbalance in cancer cells due to their altered metabolism represents a key vulnerability that can be exploited by therapeutic strategies aimed at manipulating ROS levels, with significant clinical implications by overcoming drug resistance, one of the main barriers in melanoma treatment. Furthermore, the use of miRNAs to modulate the response of tumour cells to cytotoxic treatments allows the design of personalised therapies that could optimise the efficacy of conventional treatments and reduce side effects; for example, the administration of specific miRNAs could improve the sensitivity of cancer cells to chemotherapy, representing a significant improvement in patient response. A deeper understanding of the interactions between oxidative stress, the tumour microenvironment, and miRNAs would not only help identify new therapeutic targets, but also provide innovative tools to improve clinical interventions in this cancer.

## 5. Conclusions

In this review, we have explored the involvement of microRNAs in the regulation of the signalling pathways affected by reactive oxygen species (ROS)-mediated oxidative stress in the context of melanoma. As highlighted, ROS are key intermediates in oxidation–reduction reactions, capable of modifying biomolecules and altering cellular behaviour temporarily or permanently. An imbalance in the production and scavenging of ROS, known as oxidative stress, is closely associated with the development and progression of melanoma. This phenomenon suggests that the differential modulation of oxidative stress may be associated with non-mutational epigenetic reprogramming, in which miRNAs, along with target genes and transcription factors, play a key role.

Although research on the specific interactions between reactive oxygen species and miRNAs remains limited, the evidence presented in this review suggests that these small non-coding RNAs are essential for the regulation of the signalling pathways that respond to oxidative stress. Although previous findings on miR-211, miR-21, miR-34a, Nrf2, and p53 highlight their importance in redox homeostasis and their influence on melanoma response to redox stress, the network analysis performed in this study suggests that many other molecules are involved in this process. Therefore, understanding these interactions and other variables related to redox homeostasis is crucial for the development of new diagnostic and therapeutic strategies.

The implementation of integrated regulatory network analysis models is a promising strategy. These models provide a more holistic view of the complex interactions between miRNAs, genes, and transcription factors, facilitating the identification of critical points in the signalling pathways affected by oxidative stress. Furthermore, by integrating genomic and expression data, these approaches can reveal patterns of regulation that would not be apparent if studied in isolation. This opens up the possibility of optimising clinical response and reducing treatment resistance in melanoma, providing new opportunities to develop more effective, precise, and personalised therapies.
